# Does reductive metabolism predict response to tirapazamine (SR 4233) in human non-small-cell lung cancer cell lines?

**DOI:** 10.1038/sj.bjc.6690819

**Published:** 1999-12

**Authors:** E C Chinje, A V Patterson, M P Saunders, S D Lockyer, A L Harris, I J Stratford

**Affiliations:** Experimental Oncology Group, School of Pharmacy and Pharmaceutical Sciences, University of Manchester, Oxford Road, Manchester M13 9PL, UK; ICRF Clinical Oncology Unit, University of Oxford, Churchill Hospital, Oxford, UK

**Keywords:** tirapazamine, bioreductive drug, hypoxia, NADPH:cytochrome P450 reductase, cytotoxicity, DNA-damage, lung cancer

## Abstract

The bioreductive drug tirapazamine (TPZ, SR 4233, WIN 59075) is a lead compound in a series of potent cytotoxins that selectively kill hypoxic rodent and human solid tumour cells in vitro and in vivo. Phases II and III trials have demonstrated its efficacy in combination with both fractionated radiotherapy and some chemotherapy. We have evaluated the generality of an enzyme-directed approach to TPZ toxicity by examining the importance of the one-electron reducing enzyme NADPH:cytochrome P450 reductase (P450R) in the metabolism and toxicity of this lead prodrug in a panel of seven human non-small-cell lung cancer cell lines. We relate our findings on TPZ sensitivity in these lung lines with our previously published results on TPZ sensitivity in six human breast cancer cell lines (Patterson et al (1995) *Br J Cancer* **72**: 1144–1150) and with the sensitivity of all these cell types to eight unrelated cancer chemotherapeutic agents with diverse modes of action. Our results demonstrate that P450R plays a significant role in the activation of TPZ in this panel of lung lines, which is consistent with previous observations in a panel of breast cancer cell lines (Patterson et al (1995) *Br J Cancer* **72**: 1144–1150; Patterson et al (1997) *Br J Cancer* **76**: 1338–1347). However, in the lung lines it is likely that it is the inherent ability of these cells to respond to multiple forms of DNA damage, including that arising from P450R-dependent TPZ metabolism, that underlies the ultimate expression of toxicity. © 1999 Cancer Research Campaign


*British Journal of Cancer* (1999) **81**(7), 1127–1133
© 1999 Cancer Research Campaign
Article no. bjoc.1999.0819

### Does reductive metabolism predict response to tirapazamine (SR 4233) in human non-small-cell lung cancer cell lines?

**EC Chinje[1], AV Patterson[1], MP Saunders[1], SD Lockyer[1], AL Harris[2] and IJ Stratford[1]**

[1]Experimental Oncology Group, School of Pharmacy and Pharmaceutical Sciences, University of Manchester, Oxford Road, Manchester M13 9PL, UK;
[2]ICRF Clinical Oncology Unit, University of Oxford, Churchill Hospital, Oxford, UK

**Summary** The bioreductive drug tirapazamine (TPZ, SR 4233, WIN 59075) is a lead compound in a series of potent cytotoxins that selectively kill hypoxic rodent and human solid tumour cells in vitro and in vivo. Phases II and III trials have demonstrated its efficacy in combination with both fractionated radiotherapy and some chemotherapy. We have evaluated the generality of an enzyme-directed approach to TPZ toxicity by examining the importance of the one-electron reducing enzyme NADPH:cytochrome P450 reductase (P450R) in the metabolism and toxicity of this lead prodrug in a panel of seven human non-small-cell lung cancer cell lines. We relate our findings on TPZ sensitivity in these lung lines with our previously published results on TPZ sensitivity in six human breast cancer cell lines (Patterson et al (1995) *Br J Cancer* **72**: 1144–1150) and with the sensitivity of all these cell types to eight unrelated cancer chemotherapeutic agents with diverse modes of action. Our results demonstrate that P450R plays a significant role in the activation of TPZ in this panel of lung lines, which is consistent with previous observations in a panel of breast cancer cell lines (Patterson et al (1995) *Br J Cancer* **72**: 1144–1150; Patterson et al (1997) *Br J Cancer* **76**: 1338–1347). However, in the lung lines it is likely that it is the inherent ability of these cells to respond to multiple forms of DNA damage, including that arising from P450R-dependent TPZ metabolism, that underlies the ultimate expression of toxicity.
© 1999 Cancer Research Campaign

**Keywords:** tirapazamine; bioreductive drug; hypoxia; NADPH:cytochrome P450 reductase; cytotoxicity; DNA-damage; lung cancer

Tumour hypoxia is a common clinical phenomenon that is believed to predispose to failure of treatments with radiotherapy and some chemotherapy (Gatenby et al, 1991; Hockel et al, 1993; Okunieff et al, 1993; Teicher, 1994; Nordsmark et al, 1996). This unique biochemistry of the tumour microenvironment can be exploited for therapeutic gain – through the reductive activation of prodrugs under low oxygen tension. Bioreductive drugs are selectively metabolized in tissues that experience low oxygen tensions, yielding products that are substantially more cytotoxic than their parent compounds (Adams and Stratford, 1986, 1994; Stratford and Stephens, 1989).

Tirapazamine (TPZ, 3-amino-1,2,4-benzotriazine 1,4-di-N-oxide, SR4233, WIN 59075) is the lead compound in a series of potent cytotoxins that selectively kill hypoxic rodent and human tumour cells both in vitro and in vivo (Zeman et al, 1986; Durand, 1994; Kim and Brown, 1994). Unlike other classes of bioreductive agents that can require severe radiobiological hypoxia (≤ 0.3% $pO_2$) to generate significant differential cytotoxicities (e.g. quinones and nitro(hetero)arenes), TPZ toxicity appears to extend over a broader range of ‘intermediate’ oxygen concentrations. This ensures that a greater proportion of a heterogeneous tumour cell population is effectively targeted during treatment, particularly since this ‘intermediate’ hypoxic environment is considered to dominate in determining response to radiotherapy (Brown, 1993; Koch, 1993; Wouters and Brown, 1997).

*Received 12 March 1999*
*Revised 7 June 1999*
*Accepted 11 June 1999*

*Correspondence to:* EC Chinje

Preclinical studies have demonstrated the efficacy of using TPZ in combination with both fractionated radiotherapy (Brown and Lemmon, 1990, 1991) and chemotherapeutic agents (Dorie and Brown, 1993), and TPZ is currently in phase II clinical trials for use in combination with fractionated radiotherapy, and phase III trials in combination with cisplatin chemotherapy. A recently completed phase III study of TPZ combined with cisplatin for stage IIIB and IV non-small-cell lung cancer (NSCLC) has shown a doubling of overall response rates (17% to 27%) and a significant survival advantage compared to cisplatin alone (Von Pawel and Von Roemeling, 1998).

Previous studies on the metabolism of tirapazamine using mouse liver microsomes (Walton et al, 1989, 1992) and tumour cell lines (Cahill and White, 1990; Wang et al, 1993) have demonstrated that both cytochrome P450 and NADPH:cytochrome P450 reductase (P450R) contribute to the overall reduction of TPZ to its two-electron reduced product SR4317. EPR spectroscopic studies utilizing rat liver microsomes identified P450R as the major hepatic microsomal enzyme responsible for the reductive activation of TPZ to the one-electron nitroxide radical intermediate (Lloyd et al, 1991), which is believed to be cytotoxic in its protonated form (Costa et al, 1989; Wang et al, 1992; Brown, 1993). While the role of cytochrome P450 in the one-electron activation of TPZ is unclear (Patterson et al, 1998), P450R has been directly implicated in a number of studies (Silva and O’Brien, 1993; Patterson et al, 1995, 1997). Further, the rate of metabolism has been linked to cytotoxicity under hypoxic conditions in some but not all studies (Biedermann et al, 1991). Moreover, TPZ is reduced in purified rat liver P450R (Walton et al, 1989; Cahill and White, 1990) leading to production of strand breaks in co-incubated

**Table 1** NADPH:cytochrome P450 reductase activity and SR 4233 metabolism in a panel of NSCLC cell lines

| Cell line | Characteristics | NADPH:cyt. P450 reductase (± s.d.) (nmol cyt. c $min^{-1}$ $mg^{-1}$) | SR 4317 formation rate (± s.d.) (nmol $min^{-1}$ $mg^{-1}$) |
|---|---|---|---|
| NCI-A549 | Adenocarcinoma | 22.0 ± 2.7 | 17.32 ± 3.01 |
| NCI-H322 | Bronchio-alveolar | 18.4 ± 3.2 | 17.67 ± 1.38 |
| NCI-H358 | Bronchio-alveolar | 17.7 ± 1.0 | 17.68 ± 2.01 |
| NCI-H460 | Large-cell carcinoma | 17.6 ± 2.3 | 18.92 ± 3.62 |
| NCI-H522 | Adenocarcinoma | 15.4 ± 2.2 | 13.26 ± 0.86 |
| NCI-H647 | Adenosquamous | 9.1 ± 1.0 | 8.47 ± 1.0 |
| NCI-H226 | Squamous carcinoma | 7.3 ± 1.0 | 6.57 ± 0.80 |

plasmid DNA (Fitzsimmons et al, 1994). In a panel of human breast cancer cell lines, expression of P450 reductase has been correlated with both hypoxic toxicity and metabolism of TPZ (Patterson et al, 1995). This relationship was subsequently substantiated by transecting and stably overexpressing human P450R cDNA in the human breast cell line, MDA 231 (Patterson et al, 1997). These results unequivocally demonstrated that P450R was an important determinant of the oxic and hypoxic toxicity of TPZ in breast tumour cell lines, being consistent with the one-electron reduction of TPZ by this flavoenzyme.

Two recent studies using the lung adenocarcinoma cell line, A549 (Elwell et al, 1997; Evans et al, 1998) have, however, raised apparent uncertainties over the role of P450R in TPZ metabolism and toxicity. Elwell and colleagues (1997) adapted the A549 cell line to escalating doses of TPZ under aerobic conditions and characterized the changes associated with drug resistance. A large decrease in P450R activity was found which was not reflected in the loss of hypoxic metabolism or cytotoxicity in the adapted cell lines. Meanwhile, Evans and colleagues (1998) measured both DNA damage and TPZ metabolism in isolated nuclei from the A549 cells and demonstrated that essentially all the DNA damage produced by TPZ under hypoxia could be accounted for by intranuclear metabolism. Their findings led them to believe that the intranuclear enzyme(s) implicated in TPZ-mediated toxicity was not P450R.

Thus, while it is clearly demonstrated that P450R is important in the metabolism and toxicity of TPZ in breast cancer cells, it is important to establish the generality of this contention in the light of the findings of Elwell et al (1997) and Evans et al (1998) using A549 lung cancer cells. Therefore, we report here a study of the role of P450R in the metabolism and cytotoxicity of TPZ in a panel of NSCLC cell lines. In addition, we relate our findings on TPZ sensitivity in these lung lines with our previously published results on the TPZ sensitivity in breast cancer cells (Patterson et al, 1995) and with the sensitivity of all these cell types to other unrelated cancer chemotherapeutic agents (Houlbrook et al, 1994).

### MATERIALS AND METHODS

### Chemicals

Tirapazamine, SR4317 and SR4330 were synthesized in house using previously described methods (Seng and Ley, 1972). NADPH was purchased from Boehringer Mannheim (Lewes, UK), high-performance liquid chromatography (HPLC)-grade methanol was purchased from Merck (Lutherworth, UK). All other reagents were of analytical grade and were purchased from Sigma (Poole, UK). Tissue culture medium was obtained from Gibco-BRL and fetal calf serum (FCS) from Sigma.

### Cells and culture

Table 1 lists the seven human lung cell lines used in this work. These were obtained from the National Cancer Institute (NCI) panel. All cell lines were maintained in exponential growth phase in RPMI-1640 medium, supplemented with 2 mM glutamine and 10% (v/v) FCS.

### Preparation of cell lysates

Cells in exponential growth phase were washed twice with phosphate-buffered saline (PBS) and harvested using trypsin–EDTA. Following centrifugation at 100 ***g*** for 8 min (4°C), pellets were taken and washed in ice-cold hypotonic nuclear buffer A (10 mM HEPES/potassium hydroxide pH 7.4, 1.5 mM magnesium chloride, 10 mM potassium chloride, 0.05 mM dithiothreitol). After repelleting, cells were suspended in 1.0 ml of nuclear buffer A and allowed to stand for 10 min at 4°C. Suspensions were sonicated using a MSE Soniprep 150 for 3 × 5 s at a nominal frequency of 23 kHz and an oscillation amplitude of between 5 and 10 μm. Samples were placed on ice between each sonication. The suspensions were allowed to stand in ice for further 10 min and then centrifuged at 7800 ***g*** for 15 min at 4°C. The resulting lysate was removed and stored at –80°C until required. The protein concentration of the cell lysates was determined using the Pierce protein assay (Smith et al, 1985) using high-grade bovine serum albumin (BSA) as the standard.

### NADPH:cytochrome P450 reductase activity

NADPH:cytochrome P450 reductase activity was determined spectrophotometrically as the NADPH-dependent reduction of cytochrome c. Each incubation comprised 400 μl of the cytochrome c (final concentration 50 μM), 100 μl of 10 mM potassium cyanide (final concentration 1 mM) and 10–300 μg lysate protein (10–100 μl volume) made up to 0.98 ml with 100 mM phosphate buffer, pH 7.6. The reaction was equilibrated to 37°C and was initiated by addition of 20 μl of 10 mM NADPH to the test cuvette (final concentration 200 μM), and the rate of reduction of cytochrome c was monitored at 550 nm for 3 min against a blank without NADPH. Initial rates of reaction were based on an extinction coefficient of 21 $mM^{-1}$ $cm^{-1}$ calculated (Williams and Kamin, 1962) and expressed as nmol cytochrome c reduced $min^{-1}$ $mg^{-1}$ of cell lysate protein.

### Metabolism of tirapazamine by cell lysates

Incubations were carried out in air or under nitrogen at 37°C in 4-ml amber glass vials (Chromacol, Welwyn Garden City, UK) sealed with Subaseal (Aldrich, Gillingham, UK). The 500-μl incubation volume comprised 100 μl of cell lysate (maximum final protein concentration of 1.5 mg $ml^{-1}$), 100 μl of NADPH (5 mM dissolved in incubation buffer, giving a final incubation concentration of 1 mM), 20 μl of tirapazamine (50 mM dissolved in DMSO to give a final incubation concentration of 2 mM) and 280 μl of incubation buffer (0.2 M phosphate buffer, pH 7.4). After pre-incubation under nitrogen for 10 min, the reaction was started by

© 1999 Cancer Research Campaign

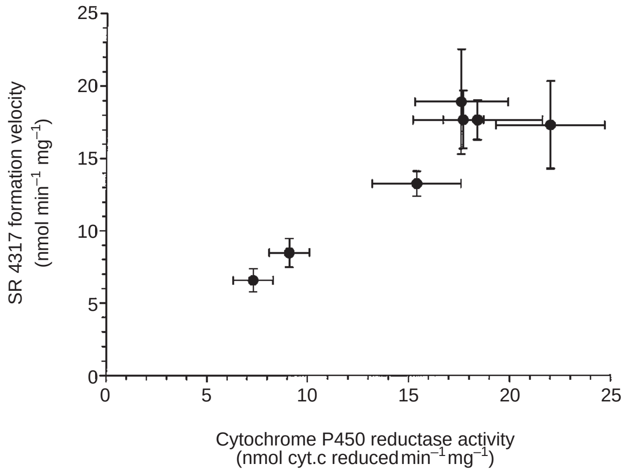

**Figure 1** A plot showing dependence on NADPH:cytochrome c (P450) reductase activity of the rate of conversion of tirapazamine under hypoxia to SR 4317 by cell lysates of the lung cancer cell lines. Bars indicate standard errors of the mean addition of tirapazamine (maintained under nitrogen) using a Hamilton syringe inserted through the Subaseal. Reactions were stopped after 40 min by transferring 2 × 200 μl aliquots of the incubate into polypropylene vials containing 50 μl of internal standard [4-nitroquinoline *N*-oxide; 0.4 mg $ml^{-1}$ in 20% (v/v) ethanol] and 400 μl of methanol. Samples were vortexed vigorously for 2 min, centrifuged for 5 min at 3000 rpm and 150 μl aliquots of the supernatants were injected onto the HPLC system for analysis. At least three lysate preparations for each cell line were incubated, each in duplicate, with duplicate analyses. Formation of SR4317 was linear for at least 40 min and up to a protein concentration of 1.5 mg $ml^{-1}$.

Concentrations of SR4317 in incubation samples were determined by isocratic reverse phase HPLC (Walton and Workman, 1990). Chromatography was performed using a waters μBondapak phenyl 4-μm radial compression cartridge in a Waters radial compression module (Waters Chromatography, Watford, UK) and protected with a phenyl guard column consisting of similar packing material. The mobile phase consisted of 32% methanol (v/v) in water delivered at a flow rate of 3 ml $min^{-1}$. Detection was at 267 nm. Approximate retention times under these conditions were 2.7, 4.6, 5.2 and 8.4 min for tirapazamine, SR4330, SR4317 and 4-nitroquinoline *N*-oxide (internal standard) respectively. Concentrations of metabolites were calculated from peak height ratios and comparison with calibration curves (0–500 μM) prepared by spiking inactivated lysate preparations with known amounts of metabolite.

### Drug sensitivity

Dose–response curves were determined using the (3-(4-5-dimethylthiazal-2-yl)-2,5-diphenyltetrazolium bromide (MTT) proliferation assay, which is based on the ability of viable cells to convert a soluble tetrazolium salt, MTT, into purple formazan crystals (Mossman, 1983). The optical density of the dissolved crystals is proportional to the number of viable cells, although this varies between cell lines as the conversion of MTT to formazan depends on the level of mitochondrial dehydrogenase activity in each cell line (Carmichael et al, 1987). The conditions for carrying out the assay have been described elsewhere (Carmichael et al, 1987; Robertson et al, 1994) and require plating $1 \times 10^3$–$5 \times 10^3$ cells (depending on cell line) into each well of a 24-well glass dish 3 h before exposure to tirapazamine for 3 h at 37°C in either air or hypoxia. The cells were then washed free of drug and allowed to grow for 4 days in 0.4 ml of fresh medium. After 4 days, MTT was added (0.2 mg $ml^{-1}$ medium) and cells were incubated for further 4 h. Culture medium and unconverted MTT were removed and the formazan crystals dissolved in 0.2 ml DMSO. An aliquot of 25 μl of glycine buffer, pH 10.5 (Plumb et al, 1989) was then added and the optical density at 540 nm measured on a multiwell spectrophotometer. Values of $IC_{50}$, the concentration of tirapazamine required to reduce the optical density by 50% compared with the untreated controls, were used as the measure of cellular sensitivity to a given treatment. The $IC_{50}$ values quoted are the means of at least three independent experiments conducted on different days.

**Table 2** Response to tirapazamine under aerobic and hypoxic conditions by NSCLC panel

| Cell line | Tirapazamine ($IC_{50}$ μM + s.d.) | | Differential toxicity[a] |
|---|---|---|---|
| | 3-h Aerobic exposure | 3-h Hypoxic exposure | |
| A549 | 247.0 ± 44 | 7.4 ± 0.5 | 33.2 |
| H322 | 980.0 ± 24 | 54.3 ± 1.7 | 18.1 |
| H358 | 1231.0 ± 62 | 48.1 ± 5.6 | 25.6 |
| H460 | 110.0 ± 10 | 4.2 ± 2.0 | 26.2 |
| H522 | 141.0 ± 12 | 8.9 ± 0.6 | 15.8 |
| H647 | 135.0 ± 22 | 6.3 ± 2.0 | 21.4 |
| H226 | 941.0 ± 189 | 22.8 ± 4.6 | 41.3 |

[a]Ratio of 3-h $IC_{50}$ values of air/nitrogen.

### Statistical analysis

The data were analysed using the standard model for a linear functional relationship with sampling errors in both variables. The data were logarithmically transformed and the pooled variance of each data set was calculated. It is assumed that the random sampling errors are normally and independently distributed with zero means and variances inversely proportional to the sample size. For statistical analysis of any two data sets, the model is fitted to the observations by the method of weighted least squares, each sample mean being weighted in direct proportion to the sample size. The statistical goodness-of-fit for any two data sets was tested by calculating the weighted mean-square deviation of the observations for mean $x_n$ and mean $y_n$ from the fitted model, and comparing this mean-square with the pooled variance within samples by a variance-ratio test. The statistical significance of the estimate of the slope of the straight line of best fit was tested by a Student's *t*-test. Tests for correlation were carried out by fitting linear functional relationship on a log-log plot, and the significance of the slope was tested by a Student's *t*-test.

### RESULTS

### Enzyme profiling and metabolism of tirapazamine

P450R activity was measured in cell lysates from the panel of seven human lung cancer cell lines (Table 1) and covered a

© 1999 Cancer Research Campaign

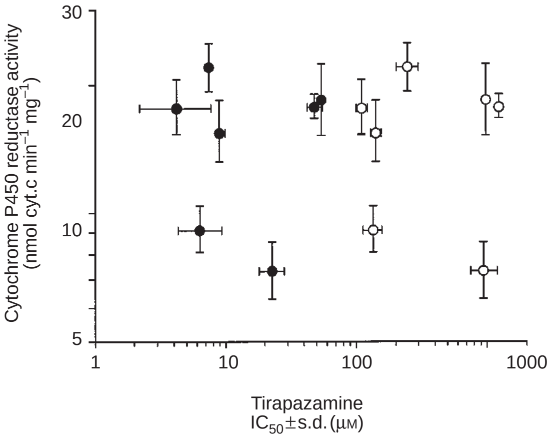

**Figure 2** Relationship between $IC_{50}$ values, derived by MTT assay of human non-small-cell lung cancer cell lines exposed to tirapazamine for 3-h under hypoxic (●) or aerobic (○) conditions and NADPH:cytochrome c (P450) reductase activity. Bars indicate standard errors of the mean

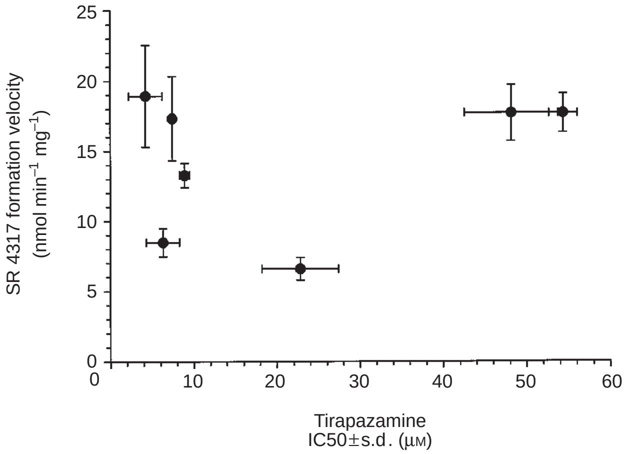

**Figure 3** Relationship between rate of conversion of tirapazamine under hypoxic conditions to SR 4317 and the $IC_{50}$ values for 3-h hypoxic exposure. Bars indicate standard errors of the mean threefold range. Table 1 includes data on the formation velocity of its two-electron reduced product SR 4317 when cell lysates were incubated with TPZ under nitrogen and in the presence of NADPH as co-factor. A plot of P450R activity versus rate of SR 4317 formation by cell lysates of each cell line, with NADPH as electron source, is shown in Figure 1. Clearly a strong correlation exists between P450R activity and SR 4317 formation, with higher values of enzyme activity resulting in greater rates of metabolism (slope = 1.03 ± 0.09; $P < 10^{-15}$). When similar experiments were carried out in air, no metabolism was detected.

### Drug sensitivity

The toxic effect of tirapazamine on the cell lines was determined by exposing cells to the drug for 3 h under aerobic and hypoxic conditions. Cytotoxicity was measured by the MTT proliferation assay and data obtained is summarized in Table 2. Also included in the Table are values of differential toxicity. Each cell shows increased sensitivity to TPZ under hypoxia, with values of differential toxicity ranging from 15 to 41. The potential role

**Table 3** Correlations between sensitivity to SR 4233 and other unrelated chemotherapeutic agents in human cancer cell lines*[a]

| Drugs | Tirapazamine exposure | | | |
|---|---|---|---|---|
| | 3-h Aerobic | | 3-h Hypoxic | |
| **Breast cancer cell lines** | | | | |
| Adriamycin | $t_4$ = –1.23 | (*P* = 0.29) | $t_4$ = 0.52 | (*P* = 0.63) |
| m-AMSA | $t_4$ = –0.47 | (*P* = 0.67) | $t_4$ = 0.15 | (*P* = 0.89) |
| Chlorambucil | $t_4$ = 0.08 | (*P* = 0.94) | $t_4$ = 0.31 | (*P* = 0.77) |
| Melphalan | $t_4$ = 1.11 | (*P* = 0.33) | $t_4$ = 1.05 | (*P* = 0.35) |
| Etoposide (VP-16) | $t_4$ = 0.33 | (*P* = 0.76) | $t_4$ = 0.45 | (*P* = 0.67) |
| BCNU | $t_4$ = 3.35 | (*P* = 0.03) | $t_4$ = 1.52 | (*P* = 0.20) |
| Cisplatin | $t_4$ = 1.42 | (*P* = 0.25) | $t_4$ = 1.66 | (*P* = 0.17) |
| Mitoxantrone | $t_4$ = 0.93 | (*P* = 0.41) | $t_4$ = 1.30 | (*P* = 0.26) |
| **Lung cancer cell lines** | | | | |
| Adriamycin | $t_5$ = 12.98 | (*P* = 00005) | $t_5$ = 7.60 | (*P* = 0.00063) |
| m-AMSA | $t_5$ = 2.44 | (*P* = 0.59) | $t_5$ = 3.42 | (*P* = 0.019) |
| Chlorambucil | $t_5$ = 8.69 | (*P* = 0.0003) | $t_5$ = 13.65 | (*P* = 0.00004) |
| Melphalan | $t_5$ = 2.44 | (*P* = 0.038) | $t_5$ = 6.62 | (*P* = 0.0012) |
| Etoposide (VP-16) | $t_5$ = 3.50 | (*P* = 0.017) | $t_5$ = 4.68 | (*P* = 0.0054) |
| BCNU | $t_5$ = 2.62 | (*P* = 0.047) | $t_5$ = 3.17 | (*P* = 0.025) |
| Cisplatin | $t_5$ = 4.55 | (*P* = 0.0061) | $t_5$ = 4.99 | (*P* = 0.0041) |
| Mitoxantrone | $t_5$ = 3.01 | (*P* = 0.03) | $t_5$ = 3.95 | (*P* = 0.011) |

[a]On 5 degrees of freedom, to ⩾ 2.57 for 5% level of significance.

of intracellular P450 reductase activity in the toxicity of TPZ across the lung line panel was assessed by examining the interrelationship between rate of NADPH-dependent cytochrome c reduction and $IC_{50}$ under acute (3-h) aerobic and hypoxic exposures. A plot showing such a dependence of $IC_{50}$ on P450R is presented in Figure 2. However, intracellular enzyme activity did not predict cytotoxicity under either aerobic (slope = –0.04 ± 0.17, $P$ = 0.80) or hypoxic (slope = –0.06 ± 0.17, $P$ = 0.73) exposure condition. Similarly, the data obtained in the present study suggest no obvious relationship between the rate at which cell lysates, obtained from the panel of lung cell lines reduced TPZ under hypoxia to SR 4317 and the $IC_{50}$ values (Figure 3).

In order to provide insights regarding the basis for this difference in TPZ toxicity between the lung lines and our previously published work on breast cancer cell lines (Patterson et al, 1995, 1997), we have compared the sensitivity of the lung and breast cell lines to a wide range of (unrelated) chemotherapeutic agents (Houlbrook et al, 1994). This latter data showed wide variations in $IC_{50}$ values for all the drugs tested, possibly reflecting the diverse range of histological cell types represented within this panel. Indeed for many chemotherapeutic agents, greater than tenfold variability in sensitivity was observed between the cell lines.

Compare-analysis of acute (3-h) aerobic and hypoxic TPZ sensitivity to each of the other agents in the breast and lung cancer cell lines is presented in Table 3. The panel of lung cancer cell lines showed significant correlation between sensitivity to TPZ and each of the members of the panel of chemotherapeutic agents. In contrast, no correlation was observed between any of the chemotherapeutic agents in the panel of human breast cancer cell lines. These results demonstrate the major inherent differences between the panel of lung and breast cell lines (Patterson et al, 1995) with respect to the response to diverse cytotoxic drugs.

### DISCUSSION

The cytotoxicity of TPZ is thought to arise as a consequence of activation by reductive enzymes that donate single electrons to the

 *© 1999 Cancer Research Campaign*

parent drug to produce radical species that cause DNA single-strand breaks (SSBs), double-strand breaks (DSBs) and, ultimately, chromosome aberrations (Brown, 1993). The present investigation examined the role of the flavoenzyme P450R in the hypoxic metabolism of TPZ across a panel of lung cancer cell line lysates. The data clearly demonstrated that intracellular P450R activity correlates strongly with the rate of formation of SR 4317, an indirect measure of radical formation ($P < 10^{-15}$). This observation is consistent with earlier reports where a good correlation was found between P450R activity and SR 4317 formation velocity ($P = 0.009$) in a panel of six human breast cancer cell lines in vitro (Patterson et al, 1995). This was unequivocally substantiated in subsequent stable expression studies with human P450R cDNA (Patterson et al, 1997). In agreement, numerous metabolism studies have implicated P450R in the reduction of TPZ to SR 4317 (Cahill and White, 1990; Walton and Workman, 1990; Riley and Workman, 1992; Walton et al, 1992; Riley et al, 1993; Silva and O'Brien, 1993). It has been suggested that fundamental differences might exist between the enzymology of human breast and lung cancer cell lines in vitro (Simm et al, 1996; Brown and Wang, 1998). However, the collective evidence would strongly suggest that the principle flavoenzyme involved in the reduction of TPZ in human cancer cell lines in vitro is P450R.

Although a strong correlation is apparent between P450R activity and SR4317 formation in the lung cell lines, cytotoxicity was apparently independent of each of these parameters, suggesting that some other factor(s) must be playing a dominant role with respect to the overall toxicity in the lung lines. Preliminary data reported elsewhere (Barham et al, 1995) and confirmed in the present studies, suggest no obvious relationship between $IC_{50}$ and the rate at which cell lysates obtained from the panel of lung cell lines reduced TPZ under hypoxia to SR 4317. This contrasts the earlier observations across the panel of human breast cancer cell lines (Patterson et al, 1995) where P450R was clearly involved in the hypoxic metabolism and cytotoxicity of TPZ.

The close relationship between TPZ activation and cytotoxicity in the panel of breast cancer cell lines derived from tumours of similar histological type suggested that a major determinant of cytotoxicity was the accumulation of TPZ-mediated DNA damage arising from P450R-dependent metabolism (Patterson et al, 1995). In contrast, the lung lines, derived from a broad range of histological subtypes, show no dependence on P450R-mediated TPZ metabolism and cytotoxicity. It is likely that this is due to the intrinsic capacity to recognize, repair and/or tolerate TPZ-mediated DNA damage which underlies the responses among the lung cell lines. It is pertinent that this distinction in chemoresponsiveness between the two panels of lung and breast cancer cell lines used has been reported from our laboratory for a spectrum of unrelated chemotherapeutic agents (Houlbrook et al, 1994). We demonstrated that the range of sensitivity for each drug tended to be greater across the lung cancer cell lines and that cross-sensitivity to these agents was common (Kendall's coefficient of concordance 0.69, $P = 0.0001$). The strong correlations between sensitivity to TPZ exposure and other unrelated chemotherapeutic agents in the lung lines but not the breast lines (Table 3) suggests that intrinsic differences may exist between the lung and breast cell lines in vitro. Indeed, the nature of the DNA damage induced by these chemotherapeutic agents is diverse, and includes intercalation, intra- and inter-strand cross-links and single and double strand-breaks, which implies a flexible response to multiple forms of DNA damage in some of the lung cell lines. Whereas TPZ sensitivity under aerobic or hypoxic exposure conditions correlated with every other drug used across the panel of lung cell lines, no relationships were apparent for any of the agents with the breast cell lines. This is consistent with the hypothesis which we propose to test, that P450R dominates response to TPZ in the breast cell lines, whereas in the NSCLC lines it is apparently their inherent sensitivity that is likely to dominate and dictate the outcome of TPZ exposure.

The role of P450R in the bioactivation of TPZ has been brought into question (Elwell et al, 1997; Evans et al, 1998). Elwell et al (1997) adapted the A549 human lung adenocarcinoma cell line to TPZ under chronic aerobic exposure condition in order to elucidate the mechanism(s) of aerobic toxicity. Their results showed that the adapted cell lines expressed extremely low levels of P450R activity (1–3% of the parental activity) but apparently only aerobic toxicity was significantly modified (9.6-fold). Hypoxic toxicity was only marginally modified (1.5-fold), which did not reflect the reduced enzyme expression. In questioning this relationship, we restored P450R activity in one of the TPZ-resistant clones by stable transfection (Chinje et al, 1998; Saunders et al, 1999). This significantly increased both hypoxic and aerobic sensitivities and restored metabolism so that it resembled that of the original parental line. Thus, P450R clearly contributes to both oxic and hypoxic TPZ-mediated toxicity although other flavoenzymes may participate in TPZ reduction (reviewed in Patterson et al, 1998).

Support for other enzyme(s) involved in TPZ activation comes from studies carried out on the role of intranuclear metabolism (Evans et al, 1998). These workers examined the co-factor requirements for TPZ metabolism leading to SSBs in isolated nuclei from A549 cells and demonstrated that DNA damage induction by TPZ had an absolute requirement for either NADPH or NADH in the nuclei. However, it was concluded from their experiments, by adding both co-factors simultaneously, that the effect was not the result of two separate enzymes with either co-factor requirement, but rather the result of an enzyme(s) capable of utilizing both co-factors as reducing equivalents. We also examined the co-factor requirements for TPZ metabolism by cell lysates derived from A549, as well as the other six from within the panel, and arrived at a similar conclusion, although NADPH supported the majority (60–90%) of the metabolism (data not shown). It is of clinical relevance to determine which other enzyme(s) may be involved in the metabolism and activation of this lead bioreductive agent.

The suggestion that other enzyme(s) requiring NADPH and/or NADH may be involved in TPZ metabolism will almost certainly place additional demands on the reducing-equivalents pool. The regulation of the pathway for producing these reducing equivalents is a critical control point since drug sensitivity as well as the ability to respond to DNA damage can be affected by their supply. NADH is formed during glycolysis following the conversion of glucose-6-phosphate to pyruvate, and NADPH is generated via the hexose monophosphate shunt (HMS) pathway. The HMS is an important biochemical pathway involved in DNA, steroid and lipid biosynthesis and in maintenance of GSH/GSSG and $NAD(P)H/NAD(P)^+$ redox equilibra. Given that the 'resting' pool size of GSH in most cell types is several-fold greater than that of NADPH, the GSH pool can serve as a redox buffer, replenishing NADPH via the reverse of the glutathione reductase reaction following drug exposure (Biaglow et al, 1977). There is also evidence to suggest that hypoxia may result in the pyridine nucleotide redox state tilting in

© 1999 Cancer Research Campaign 

favour of an increase in the availability of reducing equivalents (Jones, 1991; Arteel et al, 1998). There are reports on the stimulation of the HMS pathway in mammalian cells (including A549 cells) following incubations with a number of nitroheterocyclics (Varnes et al, 1984). In agreement with the above findings, it was observed that the increased demand for NADPH as a consequence of several-fold increases in P450R activity in the human breast cell lines MDA231 (Patterson et al, 1997), the lung line A549 (Chinje et al, 1998; Saunders et al, 1999) and in P450R-transfected T47D and HT1080 cells (AV Patterson et al, unpublished data), did not limit the sensitivity to TPZ exposure. For instance, in our published data on the breast cell lines, the changes in P450R were only sixfold and this resulted in approximately sixfold change in sensitivity (Patterson et al, 1995). Further, when P450R is overexpressed by 50-fold in the MDA231 breast cell line (Patterson et al, 1997), a tight correlation is sustained between P450R activity and cytotoxicity of TPZ. This strongly suggests that reducing equivalents are not rate limiting even at very high levels of P450R activity.

In conclusion, we have demonstrated that P450R plays a significant role in the activation of TPZ in a panel of lung cancer cell lines. However, in contrast to the panel of breast cancer cell lines where hypoxic metabolism of TPZ correlates with toxicity; in the lung lines, it is our hypothesis that the ability of these cells to respond to DNA damage may underly the ultimate expression of toxicity. Should this be the case, it would raise concerns over the use of the alkaline comet assay as a surrogate measure of response to TPZ in vivo (Siim et al, 1996). There is more to learn of the enzyme(s) involved in drug activation and the relevance of sub-cellular location, that could ultimately allow the identification and rational selection of patients who are likely to respond to TPZ therapy.

### ACKNOWLEDGEMENTS

This study was supported in part by grants from the MRC (IJS, AVP, SDL), ICRF (ALH) and the AICR (ECC). MPS was funded by an MRC Clinical Training Fellowship. We thank Dr M Jaffar for the synthesis of tirapazamine, SR 4317 and SR 4330. We are also grateful to Mr David Papworth (MRC Radiation and Genome Stability Unit, Harwell, Didcot) who carried out the statistical analysis.

### REFERENCES

Adams GE and Stratford IJ (1986) Hypoxia-mediated nitro-heterocyclic drugs in the radio and chemotherapy of cancer: an overview. *Biochem Pharmacol* **35**: 71–78

Adams GE and Stratford IJ (1994) Bioreductive drugs for cancer therapy: the search for tumour specificity. *Int J Radiat Oncol Biol Phys* **29**: 231–238

Arteel GE, Thurman RG and Raleigh JA (1998) Reductive metabolism of the hypoxia marker pimonidazole is regulated by oxygen tension independent of the pyridine nucleotide redox state. *Eur J Biochem* **253**: 743–750

Barham HM, Patterson AV, Chinje EC, Harris AL and Stratford IJ (1995) Sensitivity to tirapazamine (SR 4233) is determined by P450 reductase in human breast but not lung cancer cell lines. *Br J Cancer* **71**: 20

Biaglow JE, Jacobson B, Greenstock CL and Raleigh J (1977) *Mol Pharmacol* **13**: 262.

Biedermann KA, Wang J, Graham RP and Brown JM (1991) SR 4233 cytotoxicity and metabolism in DNA repair-competent and DNA repair-deficient cell cultures. *Br J Cancer* **63**: 358–362

Brown JM (1993) SR 4233 (Tirapazamine): a new anticancer drug exploiting hypoxia in solid tumours. *Br J Cancer* **67**: 1163–1170

Brown JM and Lemmon MJ (1990) Potentiation by the hypoxic cytotoxin SR 4233 of cell killing produced by fractionated irradiation of mouse tumors. *Cancer Res* **50**: 7745–7749

Brown JM and Lemmon MJ (1991) Tumor hypoxia can be exploited to preferentially sensitize tumors to fractionated irradiation. *Int J Radiat Oncol Biol Phys* **20**: 457–461

Cahill A and White INH (1990) Reductive metabolism of 3-amino-1,2,4-benzotriazine-1,4-dioxide (SR 4233) and the induction of unscheduled DNA synthesis in rat and human derived cell lines. *Carcinogenesis* **11**: 1407–1411

Carmichael J, De Graff WG, Gazdar AF, Minna JD and Mitchell JB (1987) Evaluation of a tetrazolium-based semi-automated colorimetric assay: assessment of chemosensitivity testing. *Cancer Res* **47**: 936–941

Chinje EC, Saunders MP, Patterson AV, Harris AL and Stratford IJ (1998) Using gene therapy to determine the mechanism of hypoxic activation of tirapazamine. *B J Cancer* **78**: S1, p. P27.

Costa AK, Baker MA, Brown JM and Trudell JR (1989) In vitro hepatotoxicity of SR 4233 (3-amino-1,2,4-benzotriazine-1,4-dioxide), a hypoxic cytotoxin and potential antitumor agent. *Cancer Res* **49**: 925–929

Dorie MJ and Brown JM (1993) Tumor-specific, schedule-dependent interaction between tirapazamine (SR 4233) and cisplatin. *Cancer Res* **53**: 4633–4636

Durand RE (1994) The influence of microenvironmental factors during cancer therapy. *In vivo* **8**: 691–702

Elwell JH, Siim BG, Evans JW and Brown JM (1997) Adaptation of human tumor cells to tirapazamine under aerobic conditions: Implications of increased antioxidant enzyme activity to mechanism of aerobic cytotoxicity. *Biochem Pharmacol* **54**: 249–257

Evans JW, Yudoh K, Delahoussaye YM and Brown JM (1998) Tirapazamine is metabolized to its DNA-damaging radical by intranuclear enzymes. *Cancer Res* **58**: 2098–2101

Fitzsimmons SA, Lewis AD, Riley RJ and Workman P (1994) Reduction of 3-amino-1,2,4-benzotriazine-1,4-di-N-oxide (tirapazamine, WIN 59075, SR 4233) to DNA-damaging species: a direct role for NADPH:cytochrome P450 oxidoreductase. *Carcinogenesis* **15**: 1503–1510

Gatenby RA, Kessler HB, Rosenblum JS, Coia LR, Moldofsky PJ, Hartz WH and Bbroder GJ (1988) Oxygen distribution in squamous cell carcinoma metastases and its relationship to the outcome of radiation therapy. *Int J Radiat Oncol Biol Phys* **14**: 831–838

Hockel M, Knoop C, Schlenger K, Vorndran B, Baussman E, Mitze M, Knapstein PG and Vaupel P (1993) Intratumoral $pO_2$ predicts survival in advanced cancer of the uterine cervix. *Radiother Oncol* **26**: 45–50

Houlbrook S, Kirk J, Stuart NSA, Stratford IJ, Harris AL, Pettit GR and Carmichael J (1994) Human tumour cell lines: a valuable model for evaluating new drugs and the mechanism underlying cytotoxic drug resistance. *Oncol (Life Sci Adv)* **13**: 69–76

Jones DP (1981) Hypoxia and drug metabolism. *Biochem Pharmacol* **30**: 1019–1023

Kim IH and Brown JM (1994) Reoxygenation and rehypoxiation in SCCVII mouse tumor. *Int J Radiat Oncol Biol Phys* **29**: 493–497

Koch CJ (1993) Unusual oxygen concentration dependence of toxicity of SR 4233, a hypoxic cell toxin. *Cancer Res* **53**: 3992–3997

Lloyd RV, Duling DR, Rumyantseva GV, Mason RP and Bridson PK (1991) Microsomal reduction of 3-amino-1,24-benzotriazine 1,4-dioxide to a free radical. *Mol Pharmacol* **40**: 440–445

Mossman T (1983) Rapid colorimetric assay for the cellular growth and survival: application to proliferation and cytotoxicity assays. *J Immunol Methods* **65**: 55–61

Nordsmark M, Overgaard M and Overgaard J (1996) Pretreatment oxygenation predicts radiation response in advanced squamous cell carcinoma of the head and neck. *Radiather Oncol* **41**: 31–40

Okunieff P, Hoeckel M, Dunphy EP, Schlenger K, Knoop C and Vaupel P (1993) Oxygen tension distributions are sufficient to explain the local response of human breast tumours treated with radiation alone. *Int J Radiat Oncol Biol Phys* **26**: 631–636

Patterson AV, Barham HM, Chinje EC, Adams GE, Harris AL and Stratford IJ (1995) Importance of P450 reductase activity in determining sensitivity of breast tumour cells to the bioreductive drug, tirapazamine (SR 4233). *Br J Cancer* **72**: 1144–1150

Patterson AV, Saunders MP, Chinje EC, Talbot DC, Harris AL and Stratford IJ (1997) Overexpression of human NADPH:cytochrome c (P450) reductase confers enhanced sensitivity to both tirapazamine (SR 4233) and RSU 1069. *Br J Cancer* **76**: 1338–1347

Patterson AV, Saunders MP, Chinje EC, Patterson LH and Stratford IJ (1998) Enzymology of tirapazamine metabolism: a review. *Anti-Cancer Drug Design* **13**: 541–573

Plumb JA, Milroy R and Kaye SB (1989) Effects of the pH dependence of 3-(4,5-dimethylthiazol-2yl)-2,5-diphenyltetrazolium bromide-formazan

© 1999 Cancer Research Campaign absorption on chemosensitivity determined by a novel tetrazolium-based assay. *Cancer Res* **49**: 4435–4440
Riley RJ and Workman P (1992) Enzymology of the reduction of the potent benzotriazine-di-N-oxide hypoxic cell cytotoxin SR 4233 (WIN 59075) by NAD(P)H: (quinone acceptor) oxidoreductase (EC 1.6.99.2) purified from Walker 256 rat tumour cells. *Biochem Pharmacol* **43**: 167–
Riley RJ, Hemingway SA, Graham MA and Workman P (1993) Initial characterization of the major mouse cytochrome P450 enzymes involved in the reductive metabolism of the hypoxic cytotoxin 3-amino-1,2,4-benzotriazine-1,4-di-N-oxide (tirapazamine, SR 4233, WIN 59075). *Biochem Pharmacol* **45**: 1065–
Robertson N, Haigh A, Adams GE and Stratford IJ (1994) Factors affecting sensitivity to EO9 in rodent and human tumour cells in vivo: DT-diaphorase and hypoxia. *Eur J Cancer* **30A**: 1013–1019
Saunders MP, Chinje EC, Patterson AV, Harris AL and Stratford IJ (1999) NADPH:cytochrome c (P450) reductase activates tirapazamine to restore hypoxic and oxic cytotoxicity in an aerobic resistant derivative of the A549 lung cancer cell line. (*in press*).
Seng F and Ley K (1972) Simple synthesis of 3-amino-1,24-benzotriazine-1,4-dioxide. *Angew Chem Int* **XI**: 1009–1010
Siim BG, Van Ziji PL and Brown JM (1996) Tirapazamine-induced DNA damage measured using the comet assay correlates with cytotoxicity towards hypoxic tumour cells in vitro. *Br J Cancer* **73**: 952–
Silva JM and O'Brien PJ (1993) Molecular mechanisms of SR 4233-induced hepatocyte toxicity under aerobic versus hypoxic conditions. *Br J Cancer* **68**: 484–491
Smith PK, Krohn RI, Hermanson GT, Mallia AK, Gartner FH, Provenzano MD, Fujimoto EK, Groeke NM, Olaon BJ and Klenk DC (1985) Measurement of protein using bicinchoninic acid. *Anal Biochem* **150**: 76–85
Stratford IJ and Stephens MA (1989) The differential hypoxic cytotoxicity of bioreductive agents determined in vitro by the MTT assay. *Int J Radiat Oncol Biol Phys* **16**: 973–976
Teicher BA (1994) Hypoxia and drug resistance. *Cancer and Metastasis Rev* **13**: 139–168
Varnes ME, Tuttle SW and Biaglow JE (1984) Nitroheterocycle metabolism in mammalian cells. Stimulation of the hexose monophosphate shunt. *Biochem Pharmacol* **33**: 1671–1677
Von Pawal J and Von Roemeling R (1998) Survival benefit from tirazone (tirapazamine) and cisplatin in advanced non-small cell lung cancer (NSCLC) patients: final results from the international phase III CATAPULT 1 trial. *Proc Am Soc Clin Oncol* **17**: 454a
Walton MI and Workman P (1990) Enzymology of the reductive activation of SR 4233: a novel benzotriazine di-N-oxide hypoxic cell cytotoxin. *Biochem Pharmacol* **39**: 1735–1742
Walton MI, Wolf CR and Workman P (1989) Molecular enzymology of the reductive bioactivation of hypoxic cell cytotoxins. *Int J Radiat Oncol Biol Phys* **16**: 983–986
Walton MI, Wolf CR and Workman P (1992) The role of cytochrome P450 and cytochrome P450 reductase in the reductive bioactivation of the novel benzotriazine di-N-oxide hypoxic cytotoxin 3-amino-1,2,4-benzotriazine-1, 4-dioxide (SR 4233, WIN 59075) by mouse liver. *Biochem Pharmacol* **44**: 251–259
Wang J, Biedermann KA and Brown JM (1992) Repair of DNA and chromosome breaks in cells exposed to SR 4233 under hypoxia or to ionizing radiation. *Cancer Res* **52**: 4473–4477
Wang J, Biedermann KA, Wolf CR and Brown JM (1993) Metabolism of the bioreductive cytotoxin SR 4233 by tumour cells: enzymatic studies. *Br J Cancer* **67**: 321–325
Williams CH Jr and Kamin H (1962) Microsomal triphosphopyridine nucleotide-cytochrome c reductase of liver. *J Biol Chem* **237**: 587–595
Wouters BG and Brown JM (1997) Cells at intermediate oxygen levels can be more important than the 'hypoxic fraction' in determining tumor response to fractionated radiotherapy. *Radiat Res* **147**: 541–550
Zeman EM, Brown JM, Lemmon MJ, Hirst VK and Lee WW (1986) SR4233: a new

*© 1999 Cancer Research Campaign*
